# Neurotoxicity of PM_2.5_-Bound Multi-Pollutant Mixtures in Parkinson’s Disease: Exploring the *INPP5D*/SHIP1-PI3K-AKT Axis via an Integrative Multi-Omics Study

**DOI:** 10.3390/jox16060184

**Published:** 2026-09-25

**Authors:** Lufei Chen, Chaoxian Dai, Zhenfang Wu, Yuhan Lin, Xiaoling Lin, Shenglang Chen, Mingzhi Fu, Yingqing Wang, Qinyong Ye

**Affiliations:** 1 Department of Neurology, Fujian Medical University Union Hospital, Fuzhou 350001, China; 2 Fujian Key Laboratory of Molecular Neurology, Institute of Neuroscience, Fujian Medical University, Fuzhou 350004, China; 3 Fujian Provincial Key Laboratory of Brain Aging and Neurodegenerative Diseases, School of Basic Medical Sciences, Fujian Medical University, Fuzhou 350122, China; 4 Department of Epidemiology and Health Statistics, School of Public Health, Fujian Medical University, Fuzhou 350122, China; 5 Department of Bioinformatics, Fujian Key Laboratory of Medical Bioinformatics, School of Medical Technology and Engineering, Fujian Medical University, Fuzhou 350122, China; 6 Clinical Research Center for Precision Diagnosis and Treatment of Neurological Diseases of Fujian Province, Fuzhou 350001, China

**Keywords:** PM_2.5_, parkinson’s disease, *INPP5D*, mendelian randomization, network toxicology

## Abstract

Accumulating epidemiological evidence links ambient PM_2.5_ exposure to elevated Parkinson’s disease (PD) risk, yet the neurotoxicity triggered by PM_2.5_ multi-pollutant mixtures under real co-exposure scenarios remains insufficiently elucidated. This study integrated two-sample Mendelian randomization (MR), mixture-oriented network toxicology, multi-omics bioinformatics, machine learning and in vitro cell validation to systematically uncover the causal molecular network of PD pathogenesis induced by mixed PM_2.5_ pollutants. Two-sample MR based on public GWAS datasets suggested a potential positive genetic association between PM_2.5_ exposure and increased PD susceptibility. We intersected PM_2.5_ component targets, PD risk genes and differential transcripts to obtain 195 shared genes, among which *INPP5D* (encoding SHIP1) was identified as the hub gene via LASSO and random forest algorithms. Molecular docking simulations further predicted that metal–organic pollutant complexes may exhibit stronger in-silico binding affinity toward SHIP1 than organic pollutants alone. Pathway enrichment screened the PI3K-AKT cascade as the core regulatory pathway. In SH-SY5Y cells, NIST SRM 1648a (urban particulate matter) treatment upregulated SHIP1 expression and phosphorylation, suppressed PI3K-AKT activity, and boosted neuroinflammation and apoptosis, whereas *INPP5D* knockdown rescued these pathological changes. Overall, our multi-omics and in vitro findings raise a plausible mechanistic hypothesis that NIST SRM 1648a-mimicked PM_2.5_-related pollutant mixtures may exacerbate PD-like neuronal injury through the SHIP1/PI3K-AKT signaling axis. This multi-dimensional evidence provides an analytical paradigm for mixed pollutant-induced neurodegeneration and highlights *INPP5D* as a promising intervention target for PM_2.5_-associated PD.

## 1. Introduction

Parkinson’s disease (PD) is the second-most-common neurodegenerative condition globally, surpassed only by Alzheimer’s disease. With the acceleration in global population aging and the increasing complexity of environmental exposure factors, the prevalence of PD has been rising markedly, making it the fastest-growing neurological disease at present [1]. According to epidemiological projections, the global patient population will exceed 25.2 million by 2050, and as the most populous country in the world, China will account for nearly half of the global cases by then [2]. Previous studies have shown that PD pathogenesis is highly heterogeneous, with only 5–15% of patients having definite genetic etiologies or identifiable genetic risk factors. This conclusion has been validated by multi-regional studies covering China, Europe, the Middle East, and both North and South America, suggesting that environmental factors may play a more critical role in the development and progression of sporadic PD [3,4,5]. Among environmental risk factors, epidemiological studies have demonstrated that fine particulate matter (PM_2.5_, aerodynamic diameter ≤ 2.5 μm), a major component of air pollution, correlates with an increased risk of PD [6,7]. For example, a prospective cohort study involving nearly 150,000 participants in the Pearl River Delta region of China found that individuals living in areas with an annual average PM_2.5_ concentration exceeding 50 μg/m^3^ had a 1.5-fold higher risk of developing PD, compared to those in areas with concentrations below 36 μg/m^3^ [8].

From a pathogenic mechanism perspective, PM_2.5_ contains ultrafine particles (≤0.1 μm in diameter) as one of its sub-components; these particles can cross the blood–brain barrier and directly infiltrate the central nervous system, inducing chronic neuroinflammation, oxidative stress damage, and abnormal activation of microglia. These pathological processes are precisely the core driving factors for the development of PD [9,10,11]. Therefore, systematically elucidating the causal association and underlying molecular mechanisms between PM_2.5_ exposure and PD risk will not only help clarify the impact weights of different air pollution sources on PD pathogenesis but also provide an important theoretical basis for formulating targeted environmental intervention strategies and PD prevention and control programs.

As an emerging interdisciplinary methodology that synergizes network biology with toxicology, network toxicology capitalizes on the integration of multi-omics datasets and advanced bioinformatics workflows to construct robust chemical–biological molecular interaction models. This approach facilitates the precise prediction of core targets and regulatory cascades that mediate the in vivo toxicological effects of exogenous toxicants [12]. On the other hand, machine learning techniques such as LASSO and random forest enable accurate identification and prediction of unknown data through model training, optimization, and evaluation [13]. The combined application of LASSO and random forest achieves a good balance among feature selection, model interpretability, predictive performance, and biological significance. Numerous studies have confirmed that machine learning algorithms can efficiently analyze genetic information, thereby accurately predicting disease outcomes and screening for key target genes [13].

This study aims to systematically characterize the neurotoxic network of PM_2.5_-derived multi-pollutant mixtures underlying PD pathogenesis and provide hypothesis-generating evidence that *INPP5D* acts as a central hub mediating possible joint toxic injury. Notably, *INPP5D* encodes the SHIP1 protein and acts as a key regulator of microglial neuroinflammation, implicated in neurodegenerative disorders including Alzheimer’s disease [14], yet its specific mechanistic function in PD, especially upon PM_2.5_ exposure, remains largely unexplored. The integrated application of two-sample Mendelian randomization and network toxicology analysis, together with molecular docking, gene set enrichment analysis, and in vitro cell experiments, to dissect the molecular network through which PM_2.5_ components regulate PD represents a key step in revealing the mechanisms of environmental pollution-induced PD progression. The findings of this study are expected to provide an important scientific basis for formulating targeted strategies for PD prevention and control.

## 2. Materials and Methods

### 2.1. Two-Sample Mendelian Randomization (MR) Analysis

Summary statistics for PM_2.5_ were obtained from a public genome-wide association study (GWAS) database (Accession No. ukb-b-10817, 423,796 European participants, continuous phenotype of 2010 PM_2.5_). Using PM_2.5_ as the exposure factor, we retained variants with *p*-values < 1 × 10^−5^ and performed linkage disequilibrium trimming using TwoSampleMR v0.4.2.6 software (10,000-kb window, r^2^ cutoff of 0.001). During SNP harmonization, palindromic variants with ambiguous strand orientation were excluded or adjusted to prevent strand-flip bias. For PD, the summary statistics were retrieved from publicly available GWAS datasets (Accession No. finn-b-G6_PARKINSON_INCLAVO, a dataset of European population with binary phenotype defining Parkinson’s disease, containing 2167 cases and 216,625 controls). No sample overlap existed between exposure and outcome GWAS cohorts. The TwoSampleMR package in R 4.5.2 was employed for all analyses. The inverse variance weighted (IVW) method was adopted as the primary approach to estimate causal effects. For robustness validation, a set of complementary analytical methods (MR-Egger regression, weighted median, weighted mode, simple mode) was implemented [15,16,17,18]. Sensitivity analysis (including MR-Egger regression) was also performed to detect potential horizontal pleiotropy. Steiger directionality test was conducted to verify the causal direction from PM_2.5_ to PD, and F-statistics were calculated to assess weak instrument bias. Furthermore, to enhance result visualization, forest plots and scatter plots were generated to illustrate the strength and direction of associations between PM_2.5_ and the risk of various secondary PD subtypes.

### 2.2. Screening of PD-Related Risk Genes

To compile PD-related genes, we screened three authoritative databases: Online Mendelian Inheritance in Man (OMIM), GeneCards, and the Comparative Toxicogenomics Database (CTD). Candidate PD-related genes collected from these sources were merged, and gene names were standardized using the UniProt database to generate a non-redundant list of PD-related risk genes.

### 2.3. Identification of Targets for PM_2.5_ Components

Six major hazardous components of PM_2.5_ were selected based on environmental monitoring datasets, the prior literature on analogous environmental exposures, and existing studies on PD [19,20,21,22,23,24,25]: benzene (Bz), benzo[a]pyrene (BaP), formaldehyde (FA), lead (Pb), arsenic (As), and cadmium (Cd). Human target genes for each chemical component were retrieved from the CTD, focusing specifically on genes known to interact with or be transcriptionally regulated by the respective chemicals in *Homo sapiens*. A comprehensive list of target genes was compiled for each PM_2.5_ component to support subsequent analyses.

### 2.4. Intersection and Protein–Protein Interaction (PPI) Network Analysis

To identify potential mediating genes linking PM_2.5_ exposure to PD, we performed intersection analysis between the target gene sets of each of the six PM_2.5_ components, the known PD-related risk gene set, and the PD differentially expressed genes (DEGs) from the GSE49036 cohort with a significance threshold of *p* < 0.05. Venn diagrams were employed to visualize the overlapping genes. These intersecting genes were submitted to the STRING database for the construction of a human PPI network, with a medium-to-high confidence interaction score (≥0.7) applied as the cutoff [26]. The resulting PPI network was imported into Cytoscape 3.9.1 for further analysis, and gene ranking was performed based on centrality metrics (degree and closeness) using the CytoHubba plugin 0.1. Additionally, Kyoto Encyclopedia of Genes and Genomes (KEGG) pathway enrichment analysis and Gene Ontology (GO) functional enrichment analysis of the intersecting genes were conducted using the “clusterProfiler 4.10.1”, “enrichplot 1.22.0”, “org.Hs.eg.db 3.18.0”, and “ggplot2 3.5.0” packages in R 4.5.2 software.

### 2.5. Screening of Hub Genes by Machine Learning

To further identify hub genes associated with PM_2.5_ exposure in PD pathogenesis, two machine learning algorithms, least absolute shrinkage and selection operator (LASSO) regression and random forest (RF), were applied on the same gene expression dataset. The input dataset contained gene expression profiles of 28 samples, with the binary outcome defined as PD case/control status (group). Preprocessing steps included removal of non-gene phenotypic variables and retention of normalized gene expression features. The dataset was randomly split into a training set (70% samples) and a held-out test set (30% samples), using stratified sampling to preserve the case-control ratio; all feature selection and model training were performed solely on the training set, while the test set was reserved for internal validation of model performance. LASSO regression was implemented using the “glmnet” R package (version 4.1.10). The optimal lambda value was determined via 10-fold cross-validation on the training set, and genes with non-zero regression coefficients at the optimal lambda were retained as LASSO-selected candidates. The RF model was built with the randomForest R package (version 4.7.1.2), with 1000 decision trees and mtry set to the square root of the total number of input gene features. Candidate genes from RF were selected based on a MeanDecreaseGini value > 0.2 [27]. Genes identified simultaneously by both algorithms were defined as overlapping candidate genes. Notably, the overlap of genes from LASSO and RF was used for feature screening only, not as independent validation, given that the two models were trained on an identical training dataset.

### 2.6. Molecular Docking

The SHIP1 protein structure (ID: 6XY7) [28] was obtained from the RCSB Protein Data Bank (PDB) database and used as the initial conformation for docking. The protein was prepared using AutoDock Tools 1.5.6 [29] by adding hydrogen atoms, assigning charges, defining atom types, and generating a pdbqt file. The small molecules formaldehyde, benzene, and benzo[a]pyrene were retrieved by Chemical Abstracts Service (CAS) number, and their three-dimensional conformations were optimized using the MMFF94 force field. Ligand pdbqt files were also generated with AutoDock Tools. Molecular docking was performed using AutoDock 4.6 [30], with the grid center covering the overall region of the protein and a grid spacing of 0.375 Å, while keeping other parameters at default settings. The top 20 scoring poses were selected for analysis. First, formaldehyde, benzene, and benzo[a]pyrene were individually docked with the protein. The highest-scoring complex structures of each compound bound to the protein were obtained. Next, Pb^2+^, As^3+^, and Cd^2+^ ions were separately docked into the formaldehyde–protein, benzene–protein, and benzo[a]pyrene–protein complexes. The non-hydrogen bonding parameters of metal ions were obtained from the updated AD4_parameters.dat file of AutoDock. This process generated the highest-scoring ternary complex structures containing metal ions, organic compounds and protein. Then, the metal–organic complex structures, which maintained the relative positions between metal ions and the corresponding organic molecules, were used as single ligands. These ligands were re-docked with the protein. The final docking models of metal–organic complexes and protein were obtained to examine their effects on the protein. The docking calculations were performed with computational support from Phadcalc (www.phadcalc.com). The docking results and interactions were visualized using Pymol 2.5.0 [31].

### 2.7. Single-Cell Transcriptomic Analysis

We obtained single-nucleus RNA sequencing data of postmortem midbrain tissues from Parkinson’s disease patients from the GEO database (GSE157783 [32]). The original study obtained ethical approval and participant informed consent via brain-bank resources, and all public-release data were fully de-identified. The downloaded dataset contained the UMI expression matrix, cell metadata, and gene annotations. We retained barcodes with >1500 UMIs, >1000 genes, <10% mitochondrial-encoded (mtDNA) counts, and <10% ribosomal gene counts. We then used Scrublet to identify potential multiplet barcodes and retained only those with an estimated doublet score < 0.15 for downstream analysis. GABA, CADPS2+ neurons, DaNs, Excitatory, and Inhibitory neuronal subtypes in the cell metadata were uniformly categorized as “Neurons”. The Seurat R package was used to construct the analysis object, with quality control criteria set as follows: genes were required to be expressed in at least 3 cells, and each cell was required to detect a minimum of 200 genes. The data were normalized via the Seurat workflow (NormalizeData, FindVariableFeatures, and ScaleData), and the top 2000 highly variable genes chosen for subsequent principal component analysis (PCA). A Shared Nearest Neighbor (SNN) graph was constructed using the first 25 principal components (PCs), with clustering executed via the Louvain algorithm integrated into the Seurat FindClusters function at a resolution of 1.5. Using the same 25 PCs, UMAP dimensional reduction was performed with 30 neighbors and a minimum distance of 0.3. Cluster marker genes were identified using the ROC method in the FindAllMarkers function, and cells were grouped into PD and control groups, based on sample origin. Neuronal cells were extracted for subpopulation analysis, followed by re-normalization, dimensionality reduction, and clustering at a resolution of 0.1. Neuronal subtypes were annotated according to the original cell metadata. A dot plot was generated to illustrate the expression frequency and intensity of *INPP5D* across different neuronal subpopulations.

### 2.8. Gene Set Enrichment Analysis (GSEA)

The PI3K-AKT signaling pathway gene set was retrieved from MSigDB. Gene expression matrices of PD and control groups were imported into GSEA software (v4.3.0) for enrichment analysis. Key parameters included 1000 permutations, sample grouping (PD/control) as phenotype labels, and pathway-related gene set selection. Enrichment significance was evaluated via normalized enrichment score (NES) and false discovery rate (FDR), with FDR < 0.05 defined as the threshold for significant differences.

### 2.9. Cell Culture

The human neuroblastoma SH-SY5Y cell line (Cell Bank, Chinese Academy of Sciences, Beijing, China, Catalog No. SCSP-5014) was maintained in DMEM/F12 with 10% fetal bovine serum (Gibco, Grand Island, NY, USA) at 37 °C, 5% CO_2_. Cells within 20 passages were used for all experiments. Before differentiation induction, cells were seeded and cultured to 50% confluency. SH-SY5Y cells were induced to differentiate using medium supplemented with 5 μmol/L retinoic acid (RA) and 1% fetal bovine serum. The differentiation medium was refreshed every 2 days, and the induction was maintained for 7 consecutive days. After differentiation, SH-SY5Y cells with neuron-like morphology were obtained for subsequent experiments. All in vitro experiments were performed with at least three independent biological replicates, and the vehicle group was set as the control.

### 2.10. PM_2.5_ Preparation and Stimulation

PM_2.5_ (NIST SRM 1648a, Gaithersburg, MD, USA) was first dispersed in DMSO, followed by further dilution with PBS. Briefly, 10 mg of 1648a particulate material was suspended in 0.05 mL DMSO, and then supplemented with 9.95 mL PBS to prepare a stock suspension at 1 mg/mL, which was stored at −20 °C. Before use, the mixture was sonicated for 20 min and vortexed for 1 min, followed by repeated pipetting to reduce particle aggregation. Prior to cell exposure, the stock suspension was diluted with culture medium to obtain working suspensions at the required final concentrations. The final DMSO concentration in working suspensions was controlled below the non-cytotoxic threshold. According to the official Certificate of Analysis (6 October 2020, dry-mass basis), SRM 1648a has certified mass fractions of As 115.5 ± 3.9 mg/kg, Cd 73.7 ± 2.3 mg/kg, Pb 0.655 ± 0.033%, and BaP 2.57 ± 0.10 mg/kg. Benzene and formaldehyde are gaseous volatile organic compounds and possess no certified values for this particulate-phase reference material.

### 2.11. Measurement of Cell Viability

Logarithmic growth phase cells were plated into 96-well culture plates. On the following day, the cells were exposed to different concentrations of MPP^+^ (0, 0.2, 0.4, 0.6, 0.8, 1, 1.2, 1.4, 1.6, and 2 mM, Sigma, D048, St. Louis, MO, USA) and SRM 1648a (0, 4, 8, 16, 32, 64, and 128 μg/mL), respectively. After 48 h of treatment, cells were rinsed three times with pre-warmed PBS to remove residual SRM 1648a particles. Then, 10 μL of MTT solution (Yuanye, R20228, Shanghai, China) and 100 μL of fresh culture medium were added to each well, and the plates were incubated for an additional 4 h in a cell culture incubator. The culture medium was removed, and 110 μL of formazan dissolution solution was added to each well. The plates were gently shaken on a low-speed shaker for 10 min to fully dissolve formazan crystals. Finally, the absorbance of each well was measured at 570 nm using a microplate reader.

### 2.12. siRNA Transfection

In a sterile centrifuge tube, 50 μL of Opti-MEM (Gibco, Grand Island, NY, USA) was mixed with transfection reagent TurboFect (Thermo Scientific, Waltham, MA, USA), gently pipetted, and incubated at room temperature for 5 min. Meanwhile, 50 μL of Opti-MEM was added to another sterile centrifuge tube, mixed with the si-*INPP5D* (Vigene Biosciences, Jinan, China), with a non-targeting scrambled siRNA as the negative control (si-NC), and incubated at room temperature for 5 min. The TF mixture and siRNA mixture were then gently combined, incubated at room temperature for 10 min, and immediately added to the cells for transfection. The final concentration of si-*INPP5D* in culture medium was 50 nM. Transfection was performed when cells reached 50% confluence, and culture medium was refreshed at 48 h post-transfection. The target sequences of si-*INPP5D* were as follows: sense: 5′-GGGUGUCGUUCAUGUUCAATT-3′; antisense: 5′-UUGAACAUGAACGACACCCTT-3′, targeting the human *INPP5D* transcript (NM_001017915.3, 1574–1592nt). The sequence of si-NC was 5′-UUCUCCGAACGUGUCACGUTT-3′.

### 2.13. Western Blotting

Cells were seeded in 6-well plates and stimulated with 1 mM MPP^+^ or 32 μg/mL PM_2.5_ for 48 h. Cells were washed with ice-cold PBS and lysed in RIPA buffer containing protease/phosphatase inhibitors. After sonication, lysates were centrifuged at 12,000× *g* for 10 min at 4 °C to collect supernatants, which were then quantified using the BCA protein assay. Equal amounts of protein were separated by 4–12% SDS-PAGE (Yeasen, 36269ES10, Shanghai, China) and transferred onto PVDF membranes. For a 1 h period at room temperature (RT), the membranes were blocked using 5% non-fat milk diluted in TBST, followed by overnight incubation at 4 °C with the primary antibodies: anti-p-SHIP1 (CST, #3941, 1:1000, Danvers, MA, USA), anti-SHIP1 (CST, #2728, 1:1000), anti-p-PI3K (CST, #4228, 1:1000), anti-PI3K (Proteintech, #67071-1-Ig, 1:1000, Wuhan, China), anti-p-AKT (Proteintech, #66444-1-Ig, 1:3000), anti-AKT1/3 (Proteintech, #60203-1-Ig, 1:1000), anti-IL-6 (CST, #12912, 1:1000), anti-cleaved Caspase-3 (CST, #9664, 1:1000), and anti-GAPDH (Proteintech, #60004-1-Ig, 1:50,000). After washing with TBST, the membranes were incubated with HRP-conjugated secondary antibodies (Bio-rad, #1706515, #1706516, 1:5000, Hercules, CA, USA) for 1 h at RT, and protein signals were visualized using an ECL chemiluminescence system. Statistical analysis was performed using one-way analysis of variance (ANOVA), with *p* < 0.05 considered statistically significant.

### 2.14. Statistical Analysis

Data are expressed as mean ± standard error of the mean (SEM). Statistical analyses were carried out using GraphPad Prism 9. One-way analysis of variance (ANOVA) followed by Tukey’s multiple comparisons test was applied for pairwise comparisons among multiple groups. A *p*-value less than 0.05 was defined as the threshold for statistical significance.

## 3. Results

### 3.1. Mendelian Randomization Analysis of the Association Between PM_2.5_ Exposure and PD Risk

Utilizing publicly available genome-wide association study (GWAS) summary data, a two-sample Mendelian randomization (MR) analysis was conducted to evaluate the causal relationship between the exposure and the outcome. Inverse variance weighted (IVW) MR analysis suggested a marginally significant positive causal association between genetically predicted higher exposure levels and increased risk of the outcome (OR = 1.881, 95% CI 1.005–3.521, *p* = 0.048) (Figure 1A). All included instrumental variables had F-statistics greater than 10 (Table S1), indicating sufficient genetic instrument strength and effectively ruling out weak instrument bias, thereby ensuring the reliability of the causal estimates. Multiple sensitivity analyses (MR-Egger, weighted median, weighted mode, and simple mode) did not reach statistical significance, but their effect directions were all consistent with the IVW result (Figure 1A); the effect estimates for all methods were positive and their confidence intervals overlapped (Figure 1B), further suggesting that the primary finding was not seriously biased by directional pleiotropy and was robust across different model assumptions. The Cochran-Q test detected no significant heterogeneity among the instrumental variables (*p* = 0.746); the MR-Egger intercept test (*p* = 0.889) and the MR-PRESSO global distortion test (*p* = 0.077) both detected no significant horizontal pleiotropy, further supporting the robustness of the causal estimates. The outcomes indicated that single nucleotide polymorphisms (SNPs) had heterogeneous weights. Yet, no extreme weak instrumental variables were detected overall, and the causal estimate was not dependent on any individual SNP (Figure 1C,D). Importantly, the IVW estimate reached only marginal statistical significance (*p* = 0.048), and none of the complementary MR sensitivity tests attained formal statistical significance, despite consistent directionality of effects. Collectively, these findings provide suggestive evidence for a positive causal relationship between the exposure and the outcome.

### 3.2. Screening of Target Genes Associated with PM_2.5_ Exposure in PD

We obtained a PD-related risk gene set from the GeneCards, OMIM, and CTD databases. To identify potential mediator genes linking PM_2.5_ exposure to PD, we performed an intersection analysis of the target gene sets of each of the six PM_2.5_ components, known PD risk gene sets, and differentially expressed genes from PD cohorts. The overlapping genes were visualized using a Venn diagram (Figure 2A), revealing 195 common target genes (Table S2). These target genes were used to construct a PPI network, which exhibited a highly interconnected topological structure, indicating tight functional associations among the proteins within the network. Topological feature analysis was conducted using Cytoscape software, revealing a distinct core–periphery hierarchy (Figure 2B,C). To further elucidate the biological processes through which PM_2.5_ exposure influences PD development, we employed LASSO regression and RF algorithms to identify key target genes. An initial screening of feature genes was performed using the LASSO regression model. After determining the optimal regularization parameter using the partial likelihood deviance curve and the one-standard-error (1-SE) criterion, three feature genes with non-zero coefficients were obtained (Figure 2D–F). Analysis with the RF algorithm screened the top 20 core features (Figure 2G). Three genes exhibited the highest feature importance scores. The intersection of the results from both screening methods yielded two candidate genes: *SLC9A1* and *INPP5D* (Figure 2H). Notably, these two algorithms were applied on the same training dataset; the overlapping genes were only used for candidate gene screening rather than independent validation. *INPP5D* (also known as SHIP1), a member of the phosphoinositide phosphatase family, has been reported to participate in immune regulation, neuronal survival, synaptic function, and neuroinflammation. Its dysregulation is closely associated with the disruption of the inflammatory microenvironment and neuronal damage in neurodegenerative diseases [33]. Meanwhile, the expression of this gene is markedly upregulated in PD (Figure 2I). Although *SLC9A1* was also identified as a candidate gene, existing evidence linking *SLC9A1* to PM_2_._5_-related PD pathogenesis remains limited. Given the well-documented role of *INPP5D* in neuroinflammation and neuronal injury—core pathological events in PD—together with its elevated expression in PD, we prioritized *INPP5D* for subsequent experiments to elucidate the specific mechanism by which *INPP5D* mediates the effect of PM_2_._5_ exposure on PD onset and progression.

### 3.3. Molecular Docking of PM_2.5_ Components and SHIP1

Molecular docking was performed between PM_2.5_ components and SHIP1. The docking scores (Table 1) showed that benzo[a]pyrene bound strongly to the SHIP1 protein, while benzene or formaldehyde alone exhibited weaker binding. When organic compounds coexisted with metal ions, the binding of all complexes to the protein was significantly enhanced. As shown in Figure 3, benzo[a]pyrene was mainly located in a hydrophobic cleft between the two large domains of the protein, interacting primarily through van der Waals forces and forming T-shaped π–π stacking with aromatic residues of the protein. The binding mode of benzene was similar, relying mainly on van der Waals interactions. In contrast, formaldehyde interacted with the protein predominantly through hydrogen bonds, along with van der Waals forces. The coordination patterns of metal ions varied: Pb^2+^ formed three coordination bonds with two side-chain carboxyl groups; As^3+^ formed two coordination bonds with one carboxyl group and one hydroxyl group; and Cd^2+^ formed two coordination bonds with one carboxyl group. These comparative docking analyses revealed enhanced binding stability of metal–organic complexes toward SHIP1. Metal cations neutralized negative charges within the SHIP1 binding pocket, a structural region capable of accommodating both organic aromatic toxicants and heavy metal ions, which facilitated the embedding of organic ligands. Collectively, metal ions and organic pollutants formed more stable metal–organic complexes when interacting with SHIP1. This molecular interaction offers a direct chemical mechanism accounting for exacerbated neuronal injury under real-world co-exposure to PM_2.5_ pollutant mixtures. These docking simulations are computational predictions built on parent-compound structures and do not introduce intracellular metabolic-transformation processes.

### 3.4. Single-Cell Transcriptomic Features of PD and Expression of INPP5D in Neurons

To elucidate the cellular heterogeneity of PD samples and the expression pattern of *INPP5D*, we performed UMAP dimensionality reduction analysis on single-cell transcriptomic data from PD patients and healthy controls (Figure 4A). Cells from different groups exhibited partial overlap and regional separation in the UMAP space, indicating differences in cellular composition between the PD and control groups. Cell type clustering revealed that each cell type formed distinct and well-demarcated clusters in the UMAP space (Figure 4B), verifying the reliability of cell clustering. Gene expression profiles varied among different neuronal subtypes (Figure 4C). Statistical comparison of *INPP5D* expression on the pooled total neuronal population showed no significant difference between the PD and control groups, suggesting that elevated bulk *INPP5D* signal in PD substantia nigra did not arise from neuronal transcriptional upregulation, but likely from tissue cellular compositional shifts. Further focusing on the expression characteristics of *INPP5D* across neuronal subtypes (Figure 4D), we found the highest baseline expression of *INPP5D* in excitatory neurons. Importantly, PD-vulnerable neurons (DaNs) also exhibited detectable expression of *INPP5D*. Given that these PD-vulnerable neurons possess basal *INPP5D* expression and represent the primary cell population damaged in PD, we subsequently adopted neuron-like cells to establish an in vitro PD injury model to explore whether activation of endogenous SHIP1 protein mediates neuronal injury independent of *INPP5D* transcriptional upregulation.

### 3.5. Elucidation of the Potential Mechanism by Which PM_2.5_ Exposure Promotes the Occurrence and Progression of PD

To elucidate the molecular cascade perturbed by PM_2.5_ multi-pollutant mixtures during PD pathological progression, we identified 195 co-expressed target genes linking PM_2.5_ and PD; this was followed by functional enrichment analysis. KEGG pathway enrichment analysis (Figure 5A) revealed that these co-expressed target genes were mainly enriched in pathways related to neurodegenerative diseases, cellular stress and survival regulation, and immunity and inflammation. Notably, the PI3K-AKT signaling pathway showed the most prominent enrichment score, suggesting its role as a core pathway mediating the link between PM_2.5_ exposure and PD pathology. GO functional enrichment analysis further demonstrated (Figure 5B) that the target genes were markedly enriched in biological processes associated with cellular stress response and cell fate regulation, with core terms including apoptosis, oxidative stress, and inflammatory response. This indicates that PM_2.5_ may drive PD progression by modulating these key pathological processes. To validate the expression profile of the PI3K-AKT signaling pathway in PD, we performed GSEA on samples from the PD group. The results (Figure 5C) showed a significant downregulation trend of the PI3K-AKT signaling pathway in the PD group (NES = −1.527153468, FDR = 0.0005284), indicating that the overall suppression of this pathway’s activity is closely associated with the pathogenesis and progression of PD. *INPP5D* encodes a 145 kDa membrane-associated phosphatase. Its core function is the specific hydrolysis of the 5′-phosphate group of the second messenger phosphatidylinositol (3,4,5)-trisphosphate [PI (3,4,5) P_3_], generating phosphatidylinositol (3,4)-bisphosphate [PI (3,4) P_2_], thereby negatively regulating the activation of the PI3K-AKT signaling pathway [34]. Existing studies have confirmed that increased *INPP5D* activity directly leads to reduced levels of phosphorylated AKT (p-AKT), consequently affecting the transduction of downstream signals governing cell metabolism and survival. Integrating the enrichment analysis results from this study, we hypothesize that in the PD pathological process, the abnormally high expression of *INPP5D* may be a key causative factor leading to the downregulation of PI3K-AKT pathway activity, ultimately promoting the onset and progression of PD by exacerbating neuronal damage.

### 3.6. SRM 1648a Exposure Activates the SHIP1 and Inhibits PI3K-AKT Signaling Axis to Promote Cell Apoptosis in an in Vitro PD Model

To clarify whether PM_2.5_ exposure contributes to PD via the SHIP1/PI3K-AKT signaling axis, we established an in vitro PD model for validation. For cellular exposure assays, NIST SRM 1648a urban particulate reference material was selected to mimic real-world complex PM_2.5_ exposure conditions containing a broad spectrum of co-existing toxic components. After RA induction, differentiated SH-SY5Y cells exhibited elongated cellular morphology, accompanied by upregulated expression of the neuron-specific protein microtubule-associated protein 2 (MAP2, Figure S1). Differentiated SH-SY5Y cells were treated with 0.2–2 mM MPP^+^ for 48 h, which consistently induced a decrease in cell viability. Exposure to varying concentrations of SRM 1648a also led to significant reductions in cell viability (Figure 6A,B). Based on these results, concentrations of 1 mM MPP^+^ and 32 μg/mL SRM 1648a were selected for subsequent detection of related protein indicators. Western blot analysis (Figure 6C–K) revealed that the expression of SHIP1 and its phosphorylated form (p-SHIP1) was elevated in both the MPP^+^ and SRM 1648a treatment groups. This increasing trend was further pronounced upon stimulation with both MPP^+^ and SRM 1648a, clearly indicating that SHIP1 plays a central role as a risk-regulating factor in the process by which PM_2.5_ exposure promotes PD onset and progression. Analysis of the PI3K-AKT pathway revealed that p-PI3K expression was significantly downregulated in the MPP^+^ and PM_2.5_ groups, with no intergroup difference in total PI3K. Both p-AKT and total AKT expression were decreased, and SRM 1648a stimulation further exacerbated the downregulation of these indicators. Furthermore, under combined MPP^+^ and SRM 1648a exposure, the expression of the pro-inflammatory factor IL-6 and the apoptosis-related protein cleaved Caspase-3 (c-Caspase-3) was significantly elevated. The original Western blot images are provided in File S1. In summary, high expression and activation of SHIP1 constitute a key mechanism by which SRM 1648a exacerbates PD pathological damage. This process likely involves the suppression of PI3K-AKT pathway activity and the upregulation of inflammation- and apoptosis-related proteins, thereby driving cellular injury under SRM 1648a exposure.

### 3.7. INPP5D Knockdown Protects Neuron-like Cells from SRM 1648a-Induced Neurotoxic Damage by Relieving Inhibition of the PI3K-AKT Signaling Pathway

To investigate the role of *INPP5D* in the exacerbation of PM_2.5_-induced Parkinson’s disease-related pathology, we performed siRNA-mediated *INPP5D* knockdown experiments in neurons and assessed key regulators of the PI3K-AKT signaling pathway, as well as markers of inflammation and apoptosis (Figure 7). *INPP5D* knockdown efficiency was confirmed by Western blotting, with a reduction of 22% in protein level (Figure 7A,C). Compared with the untreated control group (si-NC), the results obtained from the control group cells following combined exposure to MPP^+^ and SRM 1648a (si-NC + MPP^+^ + 1648a) were consistent with the findings from before (Figure 6C–K and Figure 7). In stark contrast, *INPP5D* gene knockdown (si-*INPP5D* + MPP^+^ + 1648a) reversed these pathological phenotypes. Knockdown of *INPP5D* rescues the expression levels of p-PI3K and p-AKT under pathological conditions, whilst the expression of IL-6 and cleaved caspase-3 decreased to levels comparable to those of the baseline si-NC group or the si-*INPP5D* group. Although total PI3K levels remained unchanged under all experimental conditions, *INPP5D* knockdown significantly restored total AKT expression, further confirming the role of *INPP5D* in regulating AKT stability or expression. Taken together, the results reveal that *INPP5D* plays a distinct pathogenic role in the exacerbation of SRM 1648a-induced PD-related pathology: upon co-exposure to SRM 1648a and MPP^+^, this gene drives neuroinflammation and apoptotic responses by inhibiting the PI3K-AKT survival pathway, whereas it does not exert these pathological effects when knocked down in isolation (Figure 7).

## 4. Discussion

This study systematically elucidated the molecular mechanisms by which PM_2.5_ components mediate the occurrence and progression of PD through regulating *INPP5D*, integrating multiple techniques, including MR, network toxicology, machine learning, molecular simulation, and cellular experiments. Our findings suggest that *INPP5D*, as a core signaling molecule, plays a critical mediating role in PM_2.5_-induced neuron damage. This work provides novel theoretical insights into the molecular networks underlying environmental pollutant-induced neurodegenerative diseases, while laying the foundation for investigating the environmental etiology of PD and developing targeted intervention strategies.

Two-sample MR provides suggestive genetic epidemiological support for a potential association, but it cannot definitively establish causal inference. From this genetic epidemiological perspective, our two-sample MR analysis yields new evidence linking long-term PM_2.5_ exposure to PD risk. This is consistent with numerous previous epidemiological observational studies reporting that chronic exposure to high concentrations of PM_2.5_ increases the risk of PD [35,36]. Research into the underlying mechanisms by which PM_2.5_ may induce PD is emerging. For instance, experimental studies have demonstrated that PM particles trigger the folding of α-synuclein in various cell models [37]. Additionally, PM particles can induce mitochondrial dysfunction and oxidative stress, leading to inflammation and alterations in the gut microbiota [38]. Furthermore, due to their large surface area-to-volume ratio, PM particles can efficiently deliver adsorbed toxic substances directly to the brain.

Cross-analysis across multiple databases is an effective approach to narrow down the range of potential functional genes. Through network toxicology analysis, we identified overlaps between target genes of key harmful components in PM_2.5_ and PD-related genes. The PPI network revealed an interconnected topological structure, suggesting that the occurrence and progression of PD are not driven by a single gene, but rather by a regulatory network formed through functional collaboration of proteins encoded by multiple genes. These genes collectively participate in key pathological processes such as dopaminergic neuron damage, α-synuclein aggregation, and oxidative stress imbalance [39].

The combined application of LASSO regression and the RF algorithm enhanced the reliability of screening key mediating genes. LASSO regression focuses on the linear association strength between genes and the “PM_2.5_ exposure-PD correlation”, while the RF algorithm is more suitable for capturing nonlinear contributions and interactions of genes. Their combined use reduces selection bias caused by a single algorithm, making the candidate status of *SLC9A1* and *INPP5D* more compelling. As a core regulatory factor of the phosphatidylinositol signaling pathway, *INPP5D* catalyzes the dephosphorylation of phosphatidylinositol phosphates. It is widely involved in biological processes such as immune cell activation, cell survival, and inflammatory responses [40]. Previous studies have verified its role in immune-related diseases and tumorigenesis [41]. Additionally, *INPP5D* is one of the highly expressed genetic risk factors in myeloid cells, and recent evidence suggests that SHIP1 may be involved in the development of AD. Specifically, studies have found that *INPP5D* gene expression is elevated in microglia of patients with advanced AD, and SHIP1 levels are correlated with microglial markers as well as amyloid plaque density. Moreover, the degree of *INPP5D* upregulation is positively associated with AD progression, implying its involvement in neuroinflammation and plaque formation [42]. Our experiments suggest that PM_2.5_ exposure significantly upregulated *INPP5D* expression in neuron-like cells, and this upregulation was positively correlated with the degree of neuronal damage. These results indicate that the abnormal activation of *INPP5D* may be a crucial initiating event in PM_2.5_-induced PD pathological progression. However, its function in environmental pollutant-induced neurodegenerative diseases remains unclear.

Molecular docking results revealed the direct interaction patterns between key PM_2.5_ components and SHIP1. Benzo[a]pyrene can embed into the catalytic domain of SHIP1 through its hydrophobic stacking interactions, while cadmium ions form coordination bonds with amino acid residues on the protein side chains. Both interactions significantly alter the spatial conformation of SHIP1 and enhance its phosphatase activity. Notably, all metal–organic multi-pollutant mixture complexes exhibited remarkably improved binding affinity to SHIP1, compared with isolated single components, suggesting possible joint binding interactions within PM_2.5_ toxic mixtures. Such joint binding may reshape SHIP1 catalytic conformation and overactivate its phosphatase function, persistently suppressing the neuroprotective PI3K-AKT pathway and exacerbating neuron inflammation and apoptosis under mixed pollutant exposure. This finding explains why real environmental PM_2.5_ mixture exposure induces more severe neurodegenerative damage than single laboratory toxicant treatment. This synergy may increase the likelihood of organic compounds accessing binding sites, thereby amplifying abnormal regulation of the phosphatidylinositol signaling pathway and accelerating the damage processes of the neurons. This finding further explains the significant neurotoxic effects of PM_2.5_ exposure in real-world environments. It should be emphasized that our docking simulations employed parent benzo[a]pyrene as input ligand. In vivo, BaP undergoes metabolic bioactivation mediated by CYP1A1/CYP1B1 enzymes to produce reactive metabolites, which are largely responsible for its biological toxicity [43]. Therefore, our docking results represent theoretical idealized ligand–protein interactions and may not recapitulate physiological conditions. Modelling metabolite–protein interactions and CYP-dependent metabolic networks is beyond the scope of the present work and constitutes important directions for future study.

Analysis of single-cell transcriptomic data revealed distinct cellular compositional differences between Parkinson’s disease samples and healthy controls, providing a basis for understanding the cellular heterogeneity of the disease. The study found detectable expression of *INPP5D* in neurons, suggesting a potential role for this gene in the functional regulation or pathological impairment of neurons. It is important to note that these results only indicate a spatial association between *INPP5D* expression changes and Parkinson’s disease pathology. The specific role of *INPP5D* in neurons remains to be clarified to enable a more comprehensive evaluation of its potential contribution to the development and progression of Parkinson’s disease.

The PI3K-AKT signaling pathway was the most significantly enriched. As a key intracellular signal transduction pathway, it is extensively involved in processes such as neuronal survival, proliferation, apoptosis, and stress response. Dysfunction of this pathway has been associated with the occurrence and progression of various neurodegenerative diseases [44,45]. As an environmental pollutant, PM_2.5_ is likely to interfere with the normal activity of this pathway, triggering downstream pathological responses-consistent with the research logic that environmental toxicants induce cellular damage by regulating core signaling pathways. In this study, PM_2.5_ exposure not only exacerbated the inhibition of the PI3K-AKT pathway but also significantly upregulated the expression of the pro-inflammatory cytokine IL-6 and the apoptosis-related protein c-Caspase-3, forming a pathological cascade of “SHIP1 activation–PI3K-AKT inhibition–enhanced inflammation and apoptosis.” This finding complements previous conclusions that PM_2.5_ damages dopaminergic neurons by inducing neuroinflammation and oxidative stress [46,47], further revealing the critical mediating role of the SHIP1/PI3K-AKT signaling axis in this pathological process.

To further elucidate the role of *INPP5D* in the entire PI3K-AKT axis, we reduced *INPP5D* expression through small interfering RNA (siRNA) knockdown. The results demonstrated that *INPP5D* knockdown relieved the inhibitory effect on the PI3K-AKT signaling pathway and restored pathway activity, and thereby protected neurons from PM_2.5_-induced neurotoxic damage. This further highlights the critical role of *INPP5D* in PM_2.5_-induced PD. The innovation of this study lies in identifying SHIP1 as a key molecular bridge linking PM_2.5_ exposure to PD pathogenesis, which fills the research gap regarding the regulation of phosphatidylinositol signaling pathways by environmental pollutants in PD progression. However, several limitations should be acknowledged: First, the core mechanistic evidence of this study is derived from cell-based experiments, and in vivo animal data are lacking. RA-differentiated SH-SY5Y cells are neuron-like cells rather than primary dopaminergic neurons. Furthermore, NIST SRM 1648a, the particulate reference material used for cellular assays, represents historical urban particulate matter from North America. Its chemical composition differs from real-world PM_2.5_ exposures experienced by PD patients; therefore, our in vitro findings should be extrapolated to human exposure scenarios with caution. Further epidemiological studies are needed to evaluate gene-exposure interactions between *INPP5D* polymorphisms and PM_2.5_ in the pathogenesis of Parkinson’s disease. Second, our mixed-pollutant model only incorporated six major toxic components and did not account for numerous trace contaminants. The combined effects of real-world pollutant mixtures may thus be more complex. Benzene and formaldehyde exist predominantly in the gas phase and are not present in the particulate fraction of SRM1648a. Nominal particulate mass concentrations were used in cell treatments, while actual concentrations of individual components in culture medium were not measured. In addition, control experiments with single-component and combined-component exposures were not performed. For these reasons, firm causal links cannot be established between the six candidate pollutants and the observed cellular toxicity. Third, the public transcriptomic datasets employed contain no individual PM_2.5_ exposure information for study subjects. Detected transcriptional alterations represent general molecular features of Parkinson’s disease and cannot be specifically attributed to PM_2.5_ exposure. Elevated *INPP5D* expression observed in bulk-tissue data may result from shifts in the proportional composition of different cell types within tissues. Fourth, static molecular docking cannot capture dynamic rearrangements of protein conformation or changes in enzymatic activity. Moreover, docking simulations were performed using parent chemical compounds without consideration of in vivo metabolic transformations. The IVW estimate from MR analysis reached only marginal significance, and all sensitivity analyses failed to meet statistical thresholds. These results provide only suggestive genetic–epidemiological evidence and cannot establish causality. The upstream and downstream regulatory networks of *INPP5D* remain incompletely characterized. Its crosstalk with PD-related genes (e.g., *SNCA*, *LRRK2*) [48], as well as relevant epigenetic regulatory mechanisms, require further investigation. Finally, siRNA-mediated interference only achieved partial knockdown, but not complete knockout, of *INPP5D*. Whether this partial knockdown affects basal cellular survival still requires further assessment.

Future studies could focus on the following directions. First, cellular experiments containing single-component and combined-pollutant exposure groups will be performed to disentangle individual toxic contributions and characterize joint-toxicity interactions among PM_2.5_-related contaminants. Second, specific *INPP5D* inhibitors will be developed, and their intervention effects on PM_2.5_-induced Parkinson’s-like pathology will be verified through in vivo experiments to evaluate their potential as therapeutic targets. Third, population-based epidemiological studies integrating multi-pollutant exposure quantification will be carried out to analyze gene–mixture interactions between *INPP5D* polymorphisms and complex PM_2.5_ pollution and validate whether *INPP5D* can serve as a sensitive biomarker for neurodegenerative risks induced by multi-contaminant co-exposure. Through multi-dimensional and multi-level research, these future investigations aim to provide more solid scientific evidence for the targeted prevention and control of PD, ultimately reducing the harm of environmental pollutants to nervous-system health.

## 5. Conclusions

This study constructed an evidence chain integrating two-sample Mendelian randomization, mixture-oriented network toxicology, multi-omics bioinformatics and in vitro functional validation, suggesting that exposure to PM_2.5_ multi-pollutant mixtures acts as a potential risk factor for PD and identifying *INPP5D*/SHIP1 as the core mediator of combined toxic neuronal injury. Mechanistically, possible joint interactions between organic and metallic toxicants within PM_2.5_ mixtures may upregulate SHIP1 expression and phosphorylation, repress the neuroprotective PI3K-AKT signaling cascade, and trigger persistent neuroinflammation and dopaminergic cell apoptosis. Knockdown of *INPP5D* effectively reverses all mixture-induced pathological alterations. This work establishes an integrated analytical framework for exploring neurodegeneration driven by complex environmental pollutant mixtures and highlights *INPP5D* as a promising therapeutic target for preventing PD among populations chronically exposed to multi-contaminant air mixtures.

## Figures and Tables

**Figure 1:**
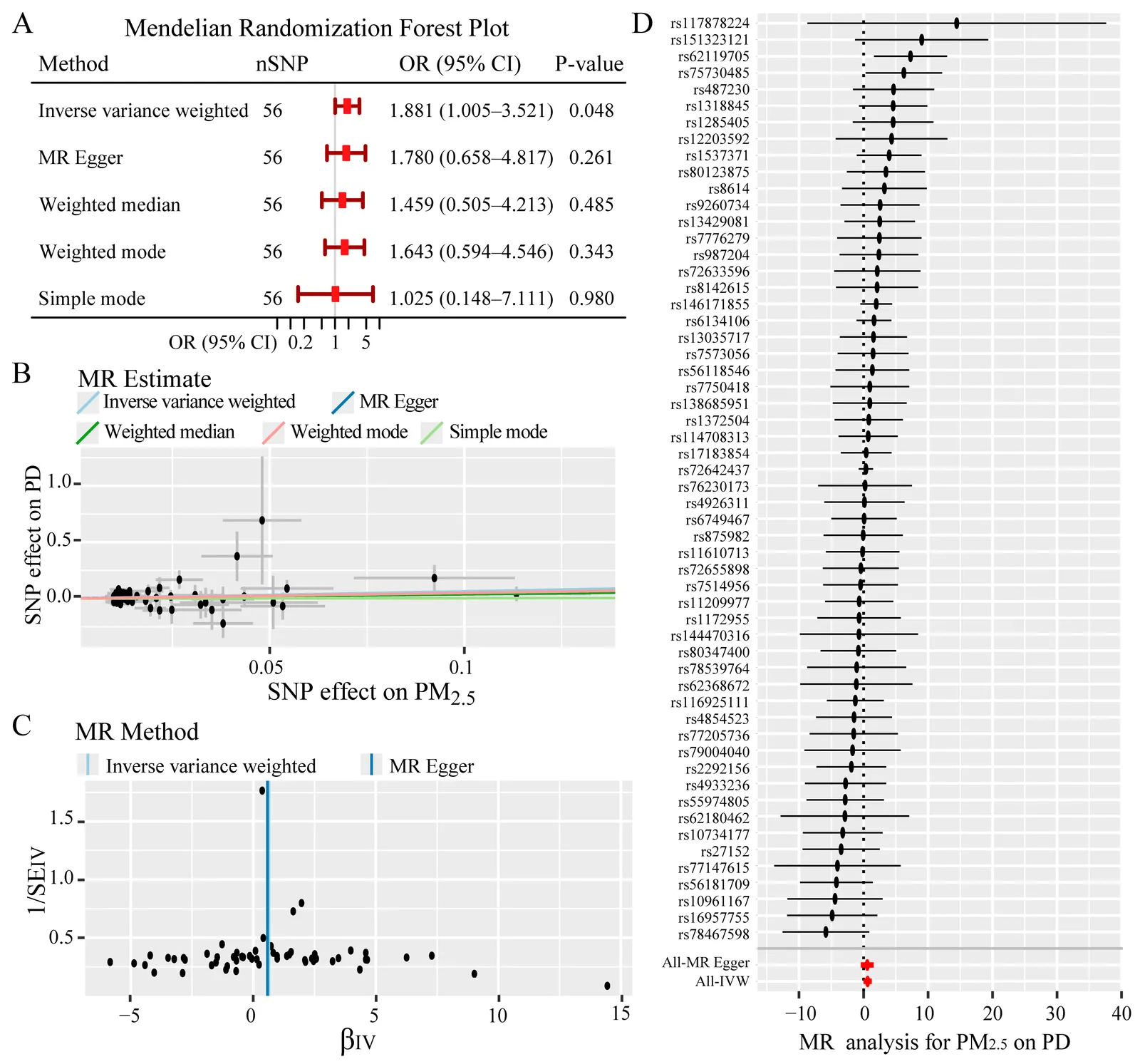
Mendelian randomization analysis of PM_2.5_ exposure and PD risk. (**A**) Forest plot of the causal association effect between PM_2.5_ and PD. (**B**) Scatter plot analysis of SNPs’ effects on PM_2.5_ and PD risk. (**C**) Scatter plot of the relationship between instrumental variable β (β_IV_) effects and weights of SNPs. (**D**) Forest plot of SNPs’ effects on MR estimates.

**Figure 2:**
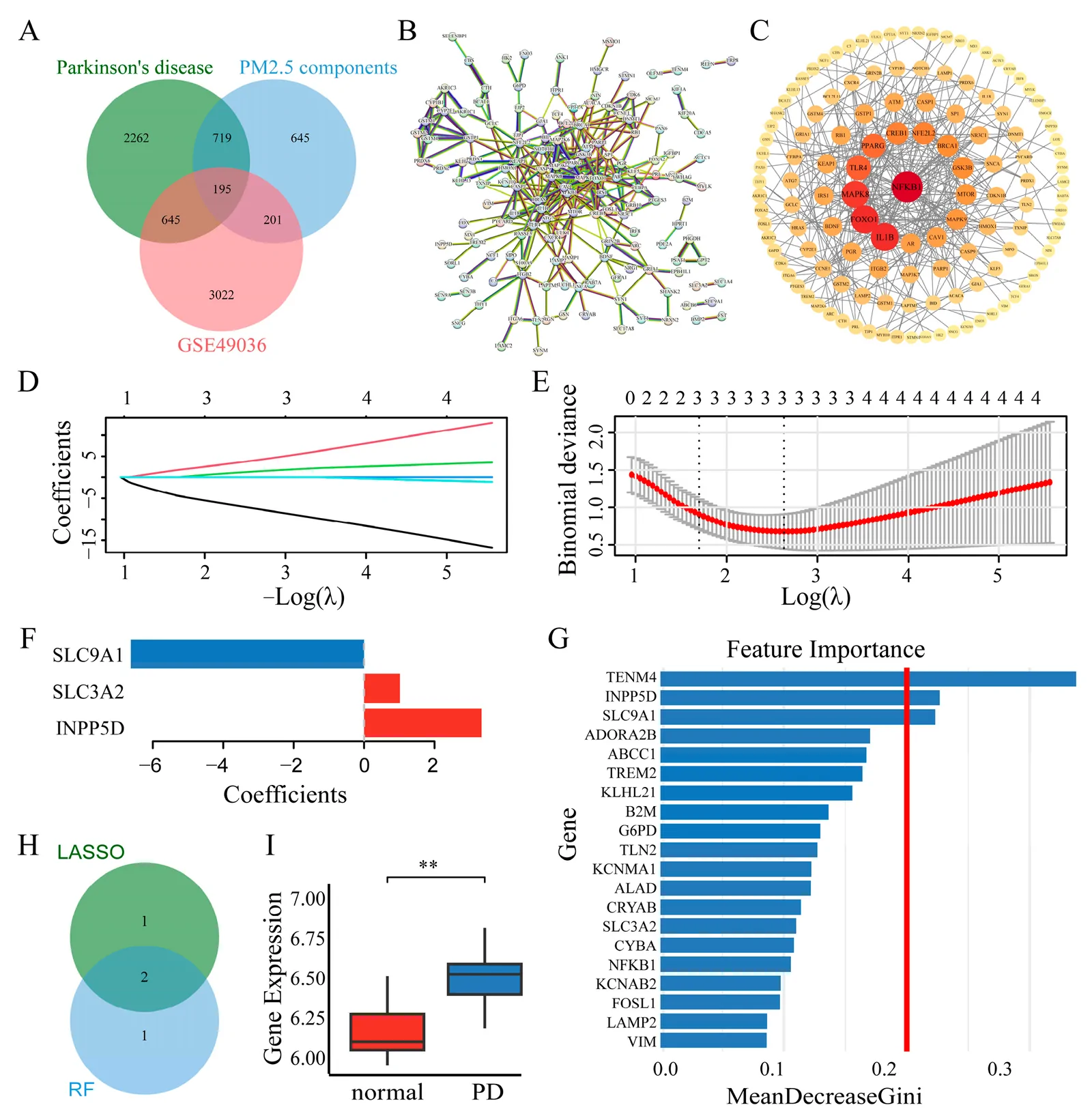
Screening of target genes associated with PM_2.5_ exposure in PD. (**A**) Venn diagram of shared molecular targets perturbed by PM_2.5_ multi-pollutant mixtures, PD risk genes and PD differentially expressed genes. (**B**) Protein–protein interaction (PPI) network associated with target gene sets. (**C**) CytoHubba analysis of the core interaction module in the target gene set. (**D**) Trajectory plot of gene coefficients as a function of −log(λ) in LASSO regression. (**E**,**F**) The curve of partial likelihood deviance versus log(λ) (solid red line), with the shaded area indicating the standard error range. The vertical line corresponds to the optimal λ value determined by the “1-SE rule”, and 3 target genes with non-zero coefficients were selected. (**G**) Feature genes identified by the RF algorithm. (**H**) Venn diagram of genes overlapping between LASSO and RF feature genes. (**I**) Expression levels of *INPP5D* in normal and PD samples from the GSE49036 dataset, ** *p* < 0.01.

**Figure 3:**
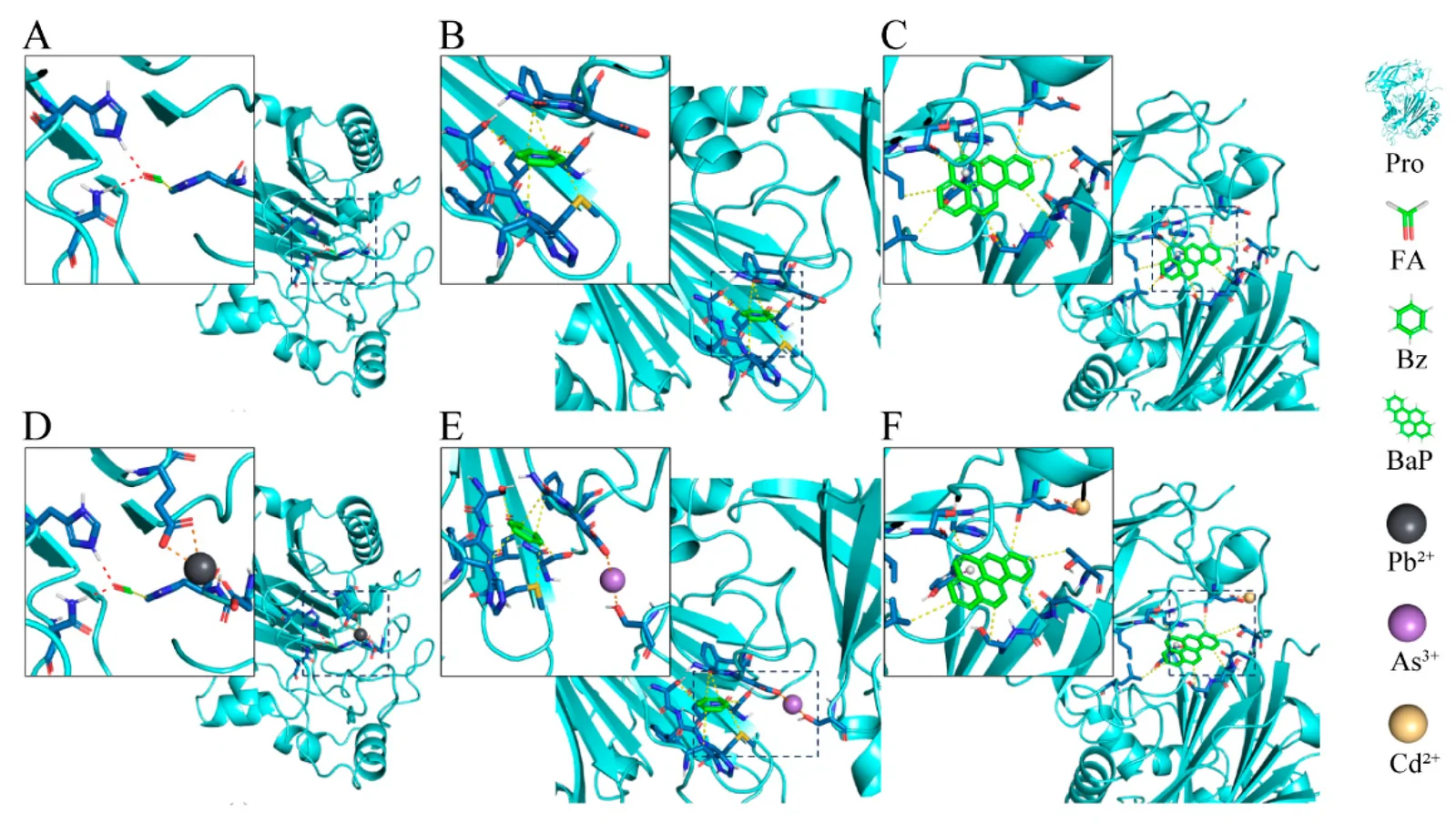
Docking models of protein–ligand. (**A**) Molecular docking visualization of SHIP1–formaldehyde complex. (**B**) Visualization of molecular docking for the SHIP1–benzene complex. (**C**) Visualization of molecular docking for the SHIP1–benzo[a]pyrene complex. (**D**) Visualization of molecular docking for the SHIP1-formaldehyde + Pb^2+^ complex. (**E**) Visualization of molecular docking for the SHIP1–benzene + As^3+^ complex. (**F**) Visualization of molecular docking for the SHIP1–benzo[a]pyrene + Cd^2+^ complex. Organic compounds are represented using a stick model. Amino acids interacting with the organic compounds are depicted in a stick model (dark color). Hydrogen bonds are shown as red dashed lines, π–π interactions as purple dashed lines, and van der Waals interactions in yellow.

**Figure 4:**
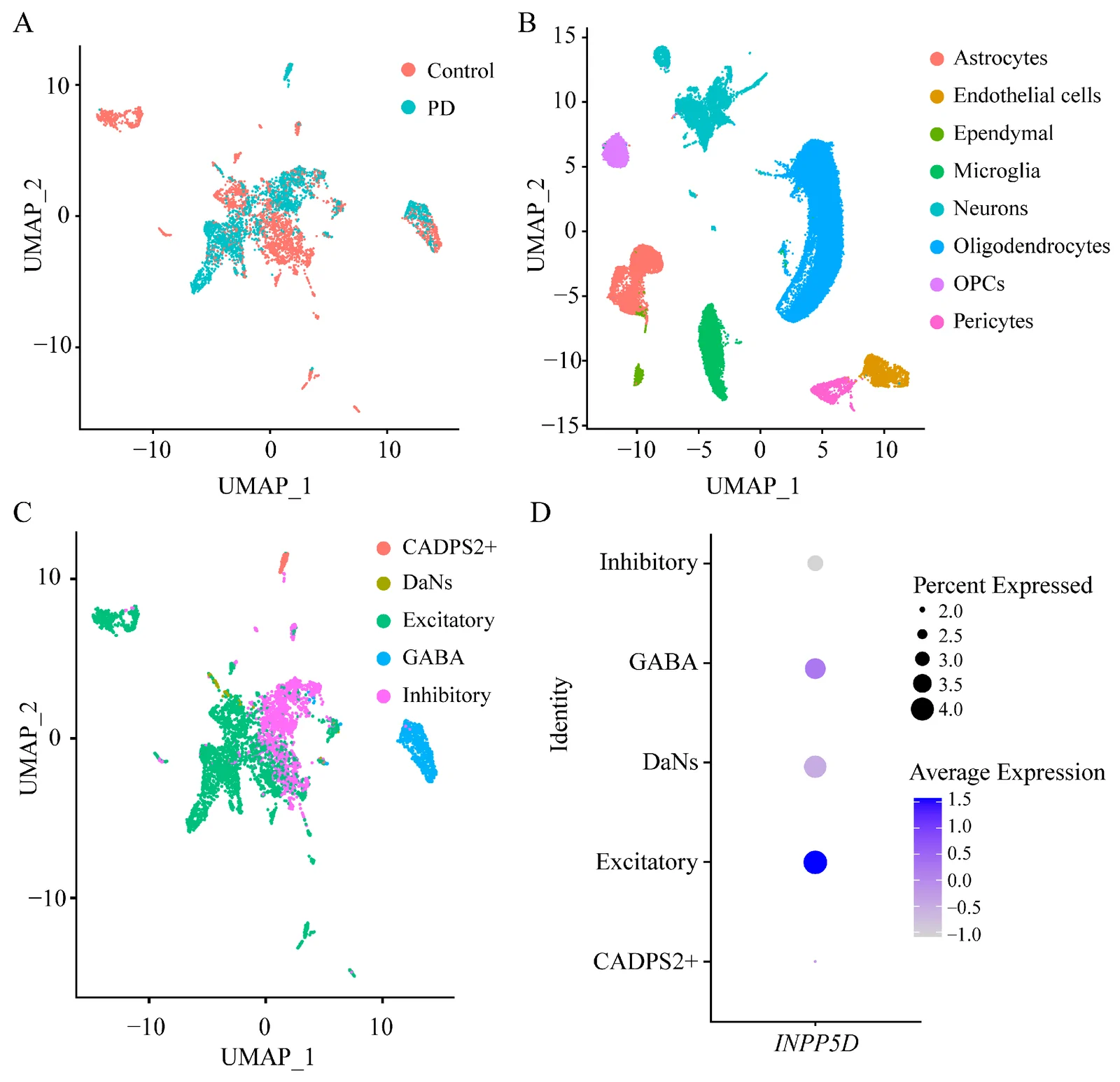
Multi-dimensional analysis of single-cell RNA sequencing data derived from Parkinson’s disease samples. (**A**) UMAP distribution of control and PD samples. (**B**) UMAP clustering of different cell types. (**C**) UMAP dimensionality reduction visualization of neuronal subtypes. (**D**) Expression profiles of *INPP5D* across neuronal subtypes.

**Figure 5:**
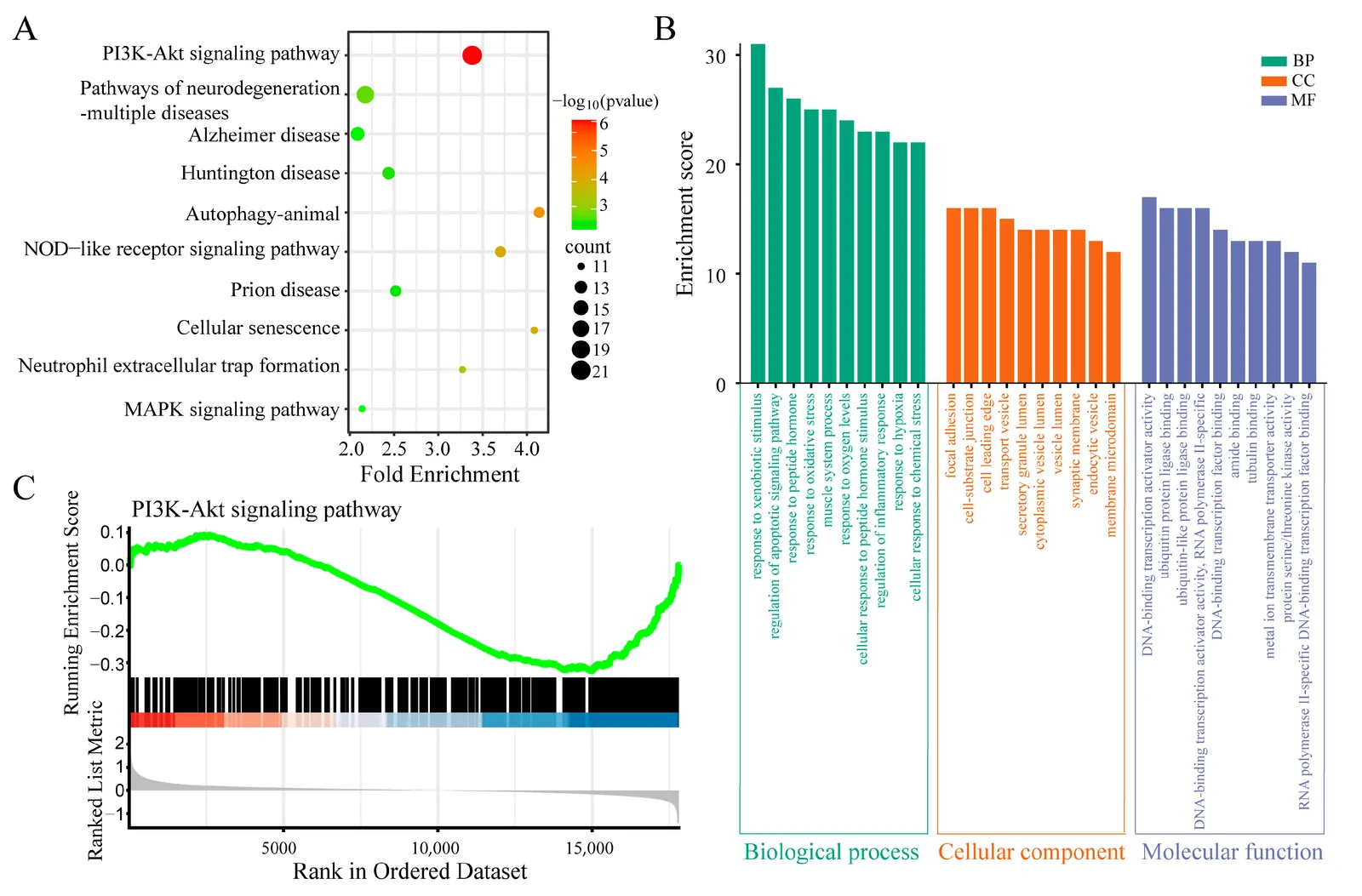
Functional enrichment of PM_2.5_-PD co-expressed target genes and expression characteristics of the PI3K-AKT pathway in PD. (**A**) Bubble plot of KEGG pathway enrichment analysis. (**B**) Categorized plot of GO functional enrichment analysis. (**C**) GSEA enrichment analysis of the PI3K-Akt signaling pathway. The green curve denotes the running enrichment score. Genes are ranked by phenotypic correlation, with red for positive correlation and blue for negative correlation. The grey area shows the ranked list metric distribution.

**Figure 6:**
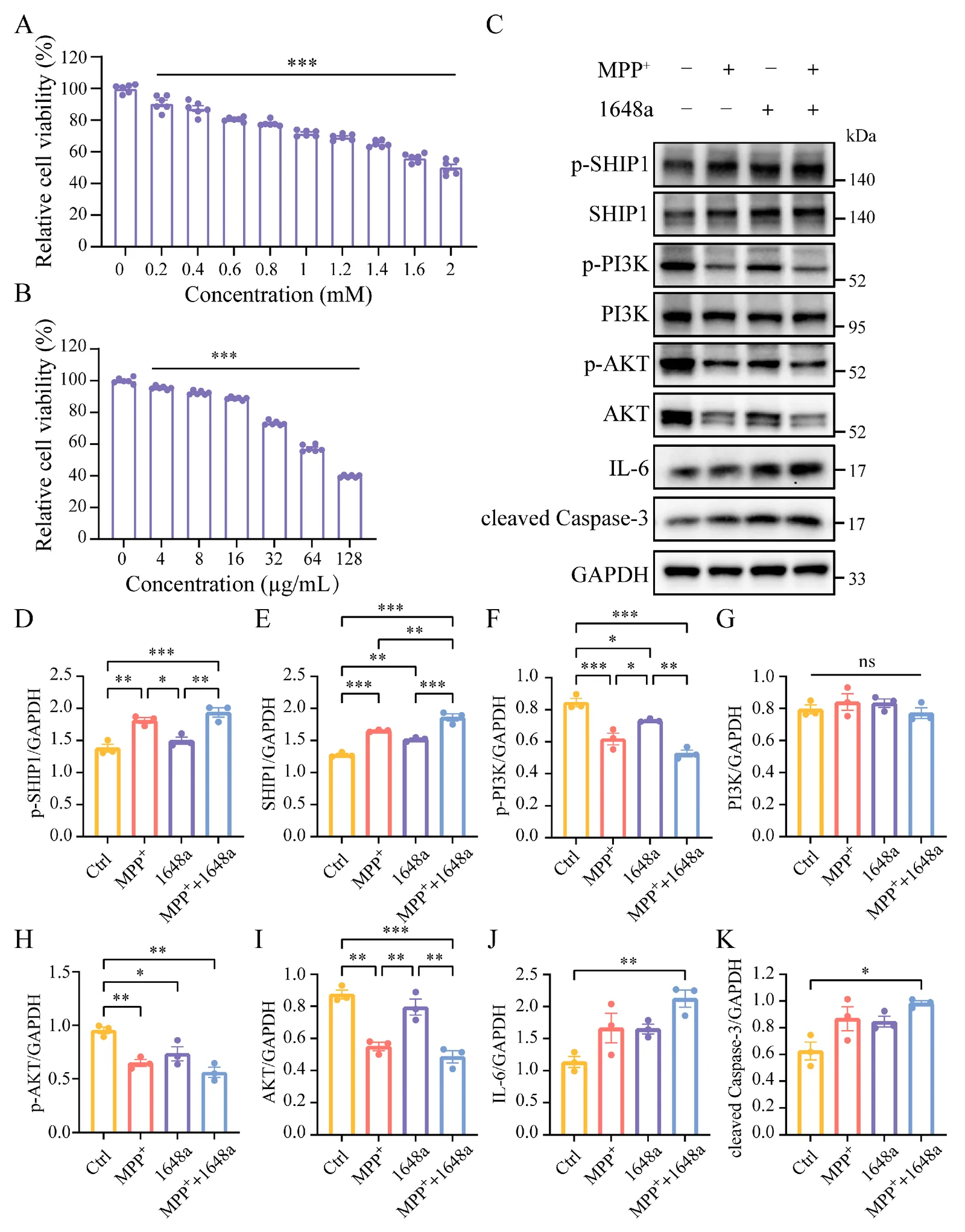
SRM 1648a exposure activates the SHIP1 and inhibits PI3K-AKT signaling axis to promote cell apoptosis in an in vitro PD model. (**A**,**B**) Cytotoxicity of MPP^+^ and SRM 1648a on cells detected by MTT assay. All data are reported as mean ± SEM (*n* = 6), with ANOVA used for statistical comparisons. *** *p* < 0.001 vs. the control group. (**C**) Western blot validation of SHIP/PI3K-AKT pathway-related proteins. (**D**–**K**) Statistical results of Western blot validation (**C**). (*n* = 3, * *p* < 0.05, ** *p* < 0.01, *** *p* < 0.001, ns, not significant).

**Figure 7:**
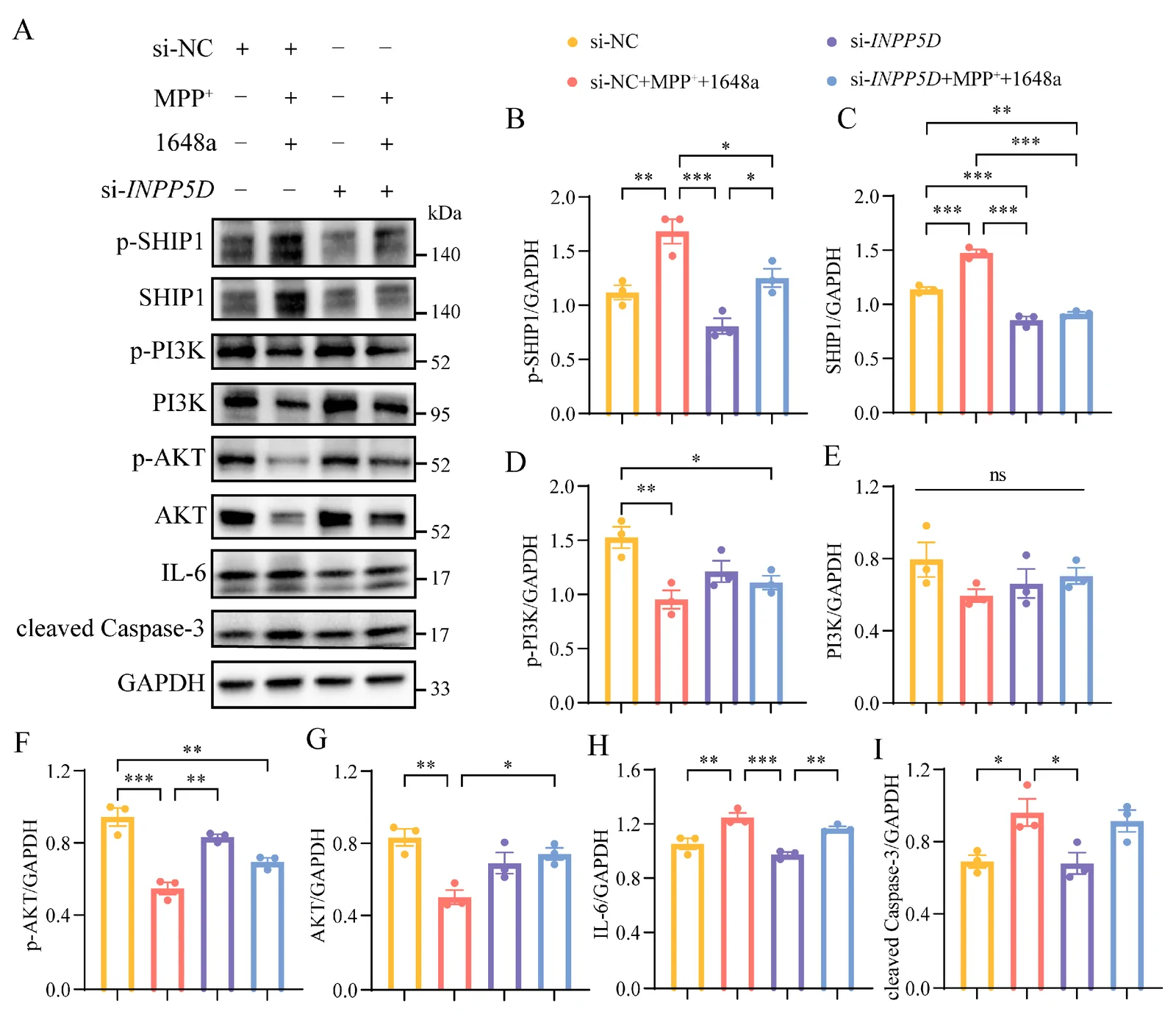
*INPP5D* knockdown alleviates SRM 1648a-induced neurotoxicity via PI3K-AKT signaling in an in vitro PD model. (**A**) Western blot validation of SHIP/PI3K-AKT pathway-related proteins following *INPP5D* knockdown. (**B**–**I**) Statistical results of Western blot validation (**A**). (*n* = 3, * *p* < 0.05, ** *p* < 0.01, *** *p* < 0.001, ns, not significant).

**Table 1:** Docking scores of protein–ligand (kcal/mol).

Model	Docking Score	Model	Docking Score
SHIP1-FA	−1.91	SHIP1-FA + Pb^2+^	−5.38
SHIP1-Bz	−3.78	SHIP1-Bz + As^3+^	−6.15
SHIP1-BaP	−8.45	SHIP1-BaP + Cd^2+^	−12.19

## Data Availability

The original contributions presented in this study are included in the Article/Supplementary Materials. Further inquiries can be directed to the corresponding authors.

## Supplementary Materials

The following supporting information can be downloaded at: https://www.mdpi.com/article/10.3390/jox16060184/s1. Table S1: F-statistics for individual SNPs used in the Mendelian-randomization analysis; Table S2: 195 common target genes; Figure S1: Immunofluorescence showing the expression of neuron-specific protein MAP2 (green) in RA-induced SH-SY5Y cells, scale bar = 50 μm; File S1: Raw blot images.

## Abbreviations

The following abbreviations are used in this manuscript:

CAS	Chemical Abstracts Service
CTD	Comparative Toxicogenomics Database
DaNs	Dopaminergic Neurons
DEGs	Differentially Expressed Genes
FDR	False Discovery Rate
GO	Gene Ontology
GSEA	Gene Set Enrichment Analysis
GWAS	Genome-wide Association Study
IVW	Inverse Variance Weighted
*INPP5D*	Inositol Polyphosphate-5-Phosphatase D
KEGG	Kyoto Encyclopedia of Genes and Genomes
MR	Mendelian Randomization
NES	Normalized Enrichment Score
OMIM	Online Mendelian Inheritance in Man
PCA	Principal Component Analysis
PCs	Principal Components
PD	Parkinson’s Disease
PDB	Protein Data Bank
PPI	Protein–Protein Interaction
RF	Random Forest
SNN	Shared Nearest Neighbor
SNPs	Single Nucleotide Polymorphisms

## References

[1] Dorsey E.R., Sherer T., Okun M.S., Bloem B.R. (2018). The Emerging Evidence of the Parkinson Pandemic. J. Park. Dis..

[2] Su D., Cui Y., He C., Yin P., Bai R., Zhu J., Lam J.S.T., Zhang J., Yan R., Zheng X. (2025). Projections for prevalence of Parkinson’s disease and its driving factors in 195 countries and territories to 2050: Modelling study of Global Burden of Disease Study 2021. BMJ.

[3] Zhao Y., Qin L., Pan H., Liu Z., Jiang L., He Y., Zeng Q., Zhou X., Zhou X., Zhou Y. (2020). The role of genetics in Parkinson’s disease: A large cohort study in Chinese mainland population. Brain.

[4] Cook L., Verbrugge J., Schwantes-An T.H., Schulze J., Foroud T., Hall A., Marder K.S., Mata I.F., Mencacci N.E., Nance M.A. (2024). Parkinson’s disease variant detection and disclosure: PD GENEration, a North American study. Brain.

[5] Westenberger A., Skrahina V., Usnich T., Beetz C., Vollstedt E.J., Laabs B.H., Paul J.J., Curado F., Skobalj S., Gaber H. (2024). Relevance of genetic testing in the gene-targeted trial era: The Rostock Parkinson’s disease study. Brain.

[6] Palacios N., Fitzgerald K.C., Hart J.E., Weisskopf M., Schwarzschild M.A., Ascherio A., Laden F. (2017). Air Pollution and Risk of Parkinson’s Disease in a Large Prospective Study of Men. Environ. Health Perspect..

[7] Cole-Hunter T., Zhang J., So R., Samoli E., Liu S., Chen J., Strak M., Wolf K., Weinmayr G., Rodopolou S. (2023). Long-term air pollution exposure and Parkinson’s disease mortality in a large pooled European cohort: An ELAPSE study. Environ. Int..

[8] Wu Q.Z., Zeng H.X., Andersson J., Oudin A., Kanninen K.M., Xu M.W., Qin S.J., Zeng Q.G., Zhao B., Zheng M. (2024). Long-term exposure to major constituents of fine particulate matter and neurodegenerative diseases: A population-based survey in the Pearl River Delta Region, China. J. Hazard. Mater..

[9] Di Domenico M., Benevenuto S.G.M., Tomasini P.P., Yariwake V.Y., de Oliveira Alves N., Rahmeier F.L., da Cruz Fernandes M., Moura D.J., Nascimento Saldiva P.H., Veras M.M. (2020). Concentrated ambient fine particulate matter (PM_2.5_) exposure induce brain damage in pre and postnatal exposed mice. Neurotoxicology.

[10] Chen J.C., Wang X., Wellenius G.A., Serre M.L., Driscoll I., Casanova R., McArdle J.J., Manson J.E., Chui H.C., Espeland M.A. (2015). Ambient air pollution and neurotoxicity on brain structure: Evidence from women’s health initiative memory study. Ann. Neurol..

[11] Cachon B.F., Firmin S., Verdin A., Ayi-Fanou L., Billet S., Cazier F., Martin P.J., Aissi F., Courcot D., Sanni A. (2014). Proinflammatory effects and oxidative stress within human bronchial epithelial cells exposed to atmospheric particulate matter (PM(2.5) and PM(>2.5)) collected from Cotonou, Benin. Environ. Pollut..

[12] Huang S. (2023). Efficient analysis of toxicity and mechanisms of environmental pollutants with network toxicology and molecular docking strategy: Acetyl tributyl citrate as an example. Sci. Total Environ..

[13] Greener J.G., Kandathil S.M., Moffat L., Jones D.T. (2022). A guide to machine learning for biologists. Nat. Rev. Mol. Cell Biol..

[14] Richardson T.I., Klein R.C., Huang K., Zhang J., Mesecar A.D., Dage J.L., Clayton B., Lamb B.T., Palkowitz A.D. (2026). Next-generation Alzheimer’s therapeutics: Target assessment and enablement at the Indiana University School of Medicine-Purdue University TREAT-AD Center. Alzheimer’s Dement..

[15] Burgess S., Small D.S., Thompson S.G. (2017). A review of instrumental variable estimators for Mendelian randomization. Stat. Methods Med. Res..

[16] Yavorska O.O., Burgess S. (2017). MendelianRandomization: An R package for performing Mendelian randomization analyses using summarized data. Int. J. Epidemiol..

[17] Burgess S., Thompson S.G. (2017). Interpreting findings from Mendelian randomization using the MR-Egger method. Eur. J. Epidemiol..

[18] Xiang M., Wang Y., Gao Z., Wang J., Chen Q., Sun Z., Liang J., Xu J. (2022). Exploring causal correlations between inflammatory cytokines and systemic lupus erythematosus: A Mendelian randomization. Front. Immunol..

[19] Li G., Yan K., Sun Y., Tian L., Wang S., Sun G. (2025). Major PM2.5 components promote intracranial aneurysm via TP53: Insights from a comprehensive network analysis. Ecotoxicol. Environ. Saf..

[20] Zhao Y., Ray A., Portengen L., Vermeulen R., Peters S. (2023). Metal Exposure and Risk of Parkinson Disease: A Systematic Review and Meta-Analysis. Am. J. Epidemiol..

[21] Xu Y., Hong H., Lin X., Tong T., Zhang J., He H., Yang L., Mao G., Hao R., Deng P. (2023). Chronic cadmium exposure induces Parkinson-like syndrome by eliciting sphingolipid disturbance and neuroinflammation in the midbrain of C57BL/6J mice. Environ. Pollut..

[22] Lee C.P., Zhu C.H., Su C.C. (2021). Increased prevalence of Parkinson’s disease in soils with high arsenic levels. Park. Relat. Disord..

[23] Sun H., Ma Z., Guo X., Chen J., He H., Tong X., Ji T., Ding S., Zhou C., Lyu Y. (2025). Plin4 modulates lipid droplet accumulation and ferroptosis in neurons exposed to benzo[a]pyrene. Cell Death Discov..

[24] Quan W., Zhang G., Li Y., Song W., Zhan J., Lin W. (2023). Upregulation of Formaldehyde in Parkinson’s Disease Found by a Near-Infrared Lysosome-Targeted Fluorescent Probe. Anal. Chem..

[25] Wang J., Ma Y., Tang L., Li D., Xie J., Sun Y., Tian Y. (2024). Long-Term Exposure to Low Concentrations of Ambient Benzene and Mortality in a National English Cohort. Am. J. Respir. Crit. Care Med..

[26] Szklarczyk D., Gable A.L., Nastou K.C., Lyon D., Kirsch R., Pyysalo S., Doncheva N.T., Legeay M., Fang T., Bork P. (2021). The STRING database in 2021: Customizable protein-protein networks, and functional characterization of user-uploaded gene/measurement sets. Nucleic Acids Res..

[27] Wang Y., Zhuang H., Jiang X.H., Zou R.H., Wang H.Y., Fan Z.N. (2023). Unveiling the key genes, environmental toxins, and drug exposures in modulating the severity of ulcerative colitis: A comprehensive analysis. Front. Immunol..

[28] Bradshaw W.J., Kennedy E.C., Moreira T., Smith L.A., Chalk R., Katis V.L., Benesch J.L.P., Brennan P.E., Murphy E.J., Gileadi O. (2024). Regulation of inositol 5-phosphatase activity by the C2 domain of SHIP1 and SHIP2. Structure.

[29] Sanner M.F. (1999). Python: A programming language for software integration and development. J. Mol. Graph. Model..

[30] Morris G.M., Huey R., Lindstrom W., Sanner M.F., Belew R.K., Goodsell D.S., Olson A.J. (2009). AutoDock4 and AutoDockTools4: Automated docking with selective receptor flexibility. J. Comput. Chem..

[31] Schrödinger L. (2020). The PyMOL Molecular Graphics System.

[32] Smajic S., Prada-Medina C.A., Landoulsi Z., Ghelfi J., Delcambre S., Dietrich C., Jarazo J., Henck J., Balachandran S., Pachchek S. (2022). Single-cell sequencing of human midbrain reveals glial activation and a Parkinson-specific neuronal state. Brain.

[33] Chou V., Pearse R.V., Aylward A.J., Ashour N., Taga M., Terzioglu G., Fujita M., Fancher S.B., Sigalov A., Benoit C.R. (2023). INPP5D regulates inflammasome activation in human microglia. Nat. Commun..

[34] Manning B.D., Toker A. (2017). AKT/PKB Signaling: Navigating the Network. Cell.

[35] Krzyzanowski B., Mullan A.F., Turcano P., Camerucci E., Bower J.H., Savica R. (2024). Air Pollution and Parkinson Disease in a Population-Based Study. JAMA Netw. Open.

[36] Zhang X., Liu H., Wu X., Jia L., Gadhave K., Wang L., Zhang K., Li H., Chen R., Kumbhar R. (2025). Lewy body dementia promotion by air pollutants. Science.

[37] Yuan X., Yang Y., Liu C., Tian Y., Xia D., Liu Z., Pan L., Xiong M., Xiong J., Meng L. (2022). Fine Particulate Matter Triggers alpha-Synuclein Fibrillization and Parkinson-like Neurodegeneration. Mov. Disord..

[38] Mutlu E.A., Comba I.Y., Cho T., Engen P.A., Yazici C., Soberanes S., Hamanaka R.B., Nigdelioglu R., Meliton A.Y., Ghio A.J. (2018). Inhalational exposure to particulate matter air pollution alters the composition of the gut microbiome. Environ. Pollut..

[39] Doostparast Torshizi A., Truong D.T., Hou L., Smets B., Whelan C.D., Li S. (2024). Proteogenomic network analysis reveals dysregulated mechanisms and potential mediators in Parkinson’s disease. Nat. Commun..

[40] Samuels J.D., Moore K.A., Ennerfelt H.E., Johnson A.M., Walsh A.E., Price R.J., Lukens J.R. (2023). The Alzheimer’s disease risk factor INPP5D restricts neuroprotective microglial responses in amyloid beta-mediated pathology. Alzheimer’s Dement..

[41] Muller S.M., Jucker M. (2024). The Functional Roles of the Src Homology 2 Domain-Containing Inositol 5-Phosphatases SHIP1 and SHIP2 in the Pathogenesis of Human Diseases. Int. J. Mol. Sci..

[42] Tsai A.P., Lin P.B., Dong C., Moutinho M., Casali B.T., Liu Y., Lamb B.T., Landreth G.E., Oblak A.L., Nho K. (2021). INPP5D expression is associated with risk for Alzheimer’s disease and induced by plaque-associated microglia. Neurobiol. Dis..

[43] Bukowska B., Mokra K., Michalowicz J. (2022). Benzo[a]pyrene-Environmental Occurrence, Human Exposure, and Mechanisms of Toxicity. Int. J. Mol. Sci..

[44] Razani E., Pourbagheri-Sigaroodi A., Safaroghli-Azar A., Zoghi A., Shanaki-Bavarsad M., Bashash D. (2021). The PI3K/Akt signaling axis in Alzheimer’s disease: A valuable target to stimulate or suppress?. Cell Stress Chaperones.

[45] AlRuwaili R., Al-Kuraishy H.M., Al-Gareeb A.I., Albuhadily A.K., Alexiou A., Papadakis M., Fetoh M.E.A., Batiha G.E. (2025). Targeting of the PI3 K/AKT/GSK3beta Pathway in Parkinson’s Disease: A Therapeutic Blueprint. Mol. Neurobiol..

[46] Du X., Guan L., Chen C., Wang X., Geng X. (2025). Long-term exposure to PM(2.5) exacerbates dopaminergic neuronal loss through CpG hypermethylation induced down-regulation of PINK1 and DJ-1 genes. Sci. Rep..

[47] Choi D., Lee G., Kim K.H., Bae H. (2022). Particulate Matter Exacerbates the Death of Dopaminergic Neurons in Parkinson’s Disease through an Inflammatory Response. Int. J. Mol. Sci..

[48] Morris H.R., Spillantini M.G., Sue C.M., Williams-Gray C.H. (2024). The pathogenesis of Parkinson’s disease. Lancet.

