# Supplementary material for: Neurotoxicity of PM2.5-Bound Multi-Pollutant Mixtures in Parkinson’s Disease: Exploring the INPP5D/SHIP1-PI3K-AKT Axis via an Integrative Multi-Omics Study
Source: J Xenobiot. 2026 Sep 25;16(6):184. doi: 10.3390/jox16060184 (PMC13705901; doi:10.3390/jox16060184)
Supplement: Supplementary file 1 [file jox-16-00184-s001.zip › jox-4531786-supplementary materials/jox-4531786-supplementary.pdf]

Neurotoxicity of $PM_{2.5}$-Bound Multi-Pollutant Mixtures in Parkinson's Disease: Exploring the *INPP5D*/SHIP1-PI3K-AKT Axis via an Integrative Multi-Omics Study

Lufei Chen , Chaoxian Dai, Zhenfang Wu, Yuhan Lin, Xiaoling Lin, Shenglang Chen, Mingzhi Fu, Yingqing Wang and Qinyong Ye

Table S1. F-statistics for individual SNPs used in the Mendelian-randomization analysis.

SNP	β	SE	F-statistic	SNP	β	SE	F-statistic
rs10734177	−0.00994	0.002198	20.46602	rs56118546	−0.01586	0.002992	28.11201
rs10961167	−0.01934	0.004066	22.62851	rs56181709	−0.01165	0.002593	20.17727
rs11209977	0.013974	0.003145	19.74065	rs6134106	0.05443	0.011897	20.93172
rs114708313	0.024558	0.004478	30.07625	rs62119705	0.011135	0.002265	24.15771
rs11610713	0.03797	0.008066	22.16165	rs62180462	−0.03511	0.007628	21.19398
rs116925111	0.053321	0.010861	24.10248	rs62368672	−0.01812	0.003929	21.25818
rs1172955	0.010736	0.002368	20.54864	rs6749467	−0.01239	0.002183	32.22849
rs117878224	−0.04804	0.010066	22.77539	rs72633596	−0.01213	0.002566	22.32576
rs12203592	0.021666	0.002591	69.91813	rs72642437	0.113396	0.019135	35.11871
rs1285405	−0.01049	0.002279	21.18565	rs72655898	−0.01132	0.002398	22.27483
rs13035717	−0.01306	0.00285	20.98541	rs7514956	−0.01298	0.00276	22.11704
rs1318845	−0.01397	0.002699	26.77719	rs75730485	−0.02677	0.006053	19.5557
rs13429081	0.013647	0.002777	24.14228	rs7573056	−0.01141	0.00217	27.6423
rs1372504	0.012291	0.002219	30.67374	rs76230173	−0.02084	0.004655	20.04397
rs138685951	−0.03082	0.006786	20.61901	rs77147615	0.024822	0.005567	19.88125
rs144470316	−0.05089	0.011465	19.70658	rs77205736	0.013522	0.002413	31.39913
rs146171855	0.092313	0.02067	19.94601	rs7750418	0.010366	0.002304	20.23526
rs151323121	−0.0416	0.0092	20.44337	rs7776279	0.010947	0.002385	21.06402
rs1537371	0.012371	0.002149	33.14877	rs78467598	−0.038	0.007686	24.44866
rs16957755	0.021686	0.004384	24.46439	rs78539764	−0.03352	0.00659	25.86871
rs17183854	−0.04343	0.00942	21.25375	rs79004040	−0.03223	0.007254	19.74059
rs2292156	0.014681	0.002878	26.02641	rs80123875	−0.01874	0.003867	23.48136
rs27152	−0.01073	0.002305	21.66471	rs80347400	0.013727	0.003034	20.46368
rs4854523	0.013445	0.002893	21.5961	rs8142615	−0.00993	0.002231	19.8177
rs487230	−0.01164	0.002512	21.44817	rs8614	0.01308	0.00279	21.98282
rs4926311	−0.01037	0.002325	19.88207	rs875982	−0.01005	0.002215	20.57337
rs4933236	0.01001	0.002202	20.65669	rs9260734	0.013983	0.003	21.72544
rs55974805	−0.01108	0.002497	19.68625	rs987204	0.010299	0.002167	22.57785

Table S2. 195 common target genes.

Gene name							
PRL	*GSTM2*	*MX1*	*DOCK4*	*PSAT1*	*KIF1A*	*BDNF*	*GSTP1*
CREB1	*SRGN*	*ADORA2B*	*PGR*	*SHANK2*	*HTRA1*	*TLR4*	*CAV1*
CASP9	*RB1*	*PHGDH*	*YWHAG*	*SCN3B*	*SYT1*	*ITPR1*	*VCAN*
GCLC	*SLC3A2*	*CCNE1*	*KIF20A*	*TXNIP*	*KCNMA1*	*HPRT1*	*NR3C1*
KEAP1	*RHBDF2*	*KLF5*	*SELENBP1*	*TLN2*	*TENM4*	*ENO3*	*GRIN2B*
STAR	*ABCC1*	*MCM7*	*GFRA1*	*CEBPA*	*PDE2A*	*HMOX1*	*COL12A1*
CPT1A	*MYH10*	*IGFBP1*	*LAMC2*	*IER5*	*BMP2*	*HRAS*	*ITGB2*
PRDX2	*GRB10*	*ABCB6*	*GPT2*	*GSTM4*	*COL11A1*	*BRCA1*	*NRG1*
IRS1	*NRXN2*	*ALAD*	*CMBL*	*KCNJ6*	*RELN*	*NFKB1*	*LOX*

BID	*UNG*	*CDKN1B*	*AKR1C1*	*OLFM1*	*ACTC1*	*DNMT1*	*ATG7*
PRDX6	*MAP2K6*	*S100A9*	*NIN*	*FOXO1*	*KCNJ10*	*PPARG*	*MAPK8*
ULK1	*ITGA6*	*DNAJB4*	*LAPTM5*	*TNFRSF19*	*PAX6*	*NCF1*	*PARP1*
MAPK9	*VIM*	*AKR1C3*	*TNFRSF21*	*SLC1A4*	*SNCG*	*G6PD*	*CASP1*
MSMO1	*ETS2*	*SYN1*	*IRF8*	*KCNAB2*	*GRHL3*	*C3*	*SLC40A1*
PYCARD	*KLHL21*	*IER3*	*CTH*	*PHLPP1*	*SNCA*	*CRYAB*	*CYP2E1*
BCL2L11	*INPP5D*	*EPB41L1*	*PTPRK*	*TTC7B*	*UCHL1*	*ADORA1*	*STMN1*
NUPR1	*LAMP1*	*FOXA2*	*PTGES3*	*RASSF5*	*LAMP2*	*GSK3B*	*SLC9A1*
ARC	*CCNG1*	*MN1*	*BCAT1*	*CDK14*	*PMP22*	*HMGCR*	*PRDX1*
TJP1	*GSN*	*MAP4K4*	*KLHL13*	*ABHD2*	*CYBA*	*IL18*	*LRP8*
ACACA	*SP1*	*AKAP12*	*ANK1*	*CDCA5*	*AR*	*CXCR4*	*MTOR*
FOSL1	*SYNM*	*MAP3K7*	*FST*	*GLA*	*NOTCH1*	*CAT*	
MYLK	*TCF4*	*NFIA*	*KRT18*	*SCN9A*	*ATM*	*NFE2L2*	
GRIA1	*EFNA1*	*MARCKSL1*	*EVL*	*TREM2*	*CBS*	*GJA1*	
CDK6	*SLC25A37*	*HK2*	*THY1*	*RAB7A*	*MPO*	*B2M*	
CYP1B1	*SLC17A8*	*TJP2*	*MTUS1*	*SORL1*	*IL1B*	*GSTM1*	

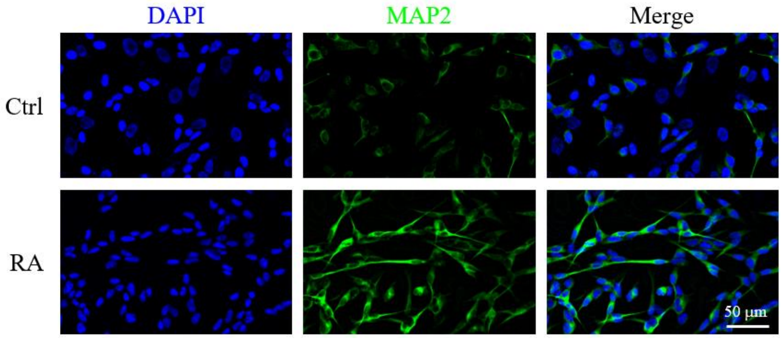

Figure S1. Immunofluorescence showing the expression of neuron-specific protein MAP2 (green) in RA-induced SH-SY5Y cells, scale bar=50 μm.
